# Parental Identity and Subjective Well-Being in Older Women: The Moderating Role of the Human–Dog Bond

**DOI:** 10.3390/bs16040567

**Published:** 2026-04-09

**Authors:** Phillipa D. Bandis, Deanna L. Tepper, Joanna Shnookal, Jemma R. Sheppard, Pauleen C. Bennett

**Affiliations:** 1 School of Psychology and Public Health, La Trobe University, Melbourne, VIC 3082, Australia; 19352078@students.latrobe.edu.au (P.D.B.); d.tepper@latrobe.edu.au (D.L.T.); j.shnookal@latrobe.edu.au (J.S.); 21214171@students.latrobe.edu.au (J.R.S.); 2 School of Psychology and Public Health, La Trobe University, Bendigo, VIC 3552, Australia

**Keywords:** parental identity, subjective well-being, human–dog bond, dog–owner relationship, older women

## Abstract

Parental identity, the extent to which individuals integrate parenting roles into their self-concept, is associated with subjective well-being (SWB). However, research has largely focused on current parents, with limited attention to those with alternative caregiving roles. Companion dogs often act as caregiving figures, but their role in shaping identity and well-being processes has not been fully explored. This cross-sectional, survey-based study examined whether parental identity is associated with SWB, regardless of parental status, and whether the human–dog bond moderates any association in older women. Women dog owners aged 40 years and over (*N* = 296, *M* age = 51.6) completed an online survey including the Parental Identity and Enjoyment Scale, the Dog Owner Relationship Scale, the Satisfaction With Life Scale, and the Flourishing Scale. Parental identity was positively associated with life satisfaction, r = 0.38, *p* < 0.001, and flourishing, r = 0.23, *p* < 0.001, and moderated regression models were significant for both (*p* < 0.001). However, interaction effects between parental identity and the human–dog bond were not significant. These findings extend identity theory, demonstrating that parental identity predicts SWB across diverse pathways and independently of parental status. The results contribute to emerging research on caregiving identities and highlight the importance of considering identity processes, rather than parental status alone, when examining well-being in older women.

## 1. Introduction

Women are navigating increasingly diverse pathways to caregiving, identity, and fulfilment, shaped by broad social changes such as declining fertility, delayed parenthood, and rising voluntary childlessness (Peterson & Engwall, 2019; Volsche et al., 2023). At the same time, alternative caregiving roles are gaining visibility, with companion animals, often described as ‘fur-babies’, providing opportunities for emotional closeness, responsibility, and role expression (Owens & Grauerholz, 2019). The human–dog bond, in particular, contributes to psychological functioning by providing social support and role continuity, and, for some individuals, may partially compensate for unmet caregiving or attachment needs (Kurdek, 2009; Merkouri et al., 2022). Research demonstrates that both identity commitments and close relationships with others, human or non-human, play a key role in shaping well-being outcomes (Piotrowski, 2021), making it imperative to research how the human–dog bond may shape emotional functioning.

Parental identity refers to the degree to which individuals internalise, reflect on, and integrate the parenting role into their broader self-concept (Fadjukoff et al., 2016; Piotrowski, 2021). Older adulthood is commonly conceptualised as a life stage spanning early to later adulthood, often characterised by reflection on life roles, identity commitments, long-term life trajectories, and, for women, important physical transitions such as menopause and post-menopause (Infurna et al., 2020). While historically studied among parents, parental identity is increasingly recognised to exist across diverse parenting experiences, including voluntary and involuntary childlessness (Laurent-Simpson, 2017). Stronger parental identity is linked to greater life satisfaction (Piotrowski, 2021), whereas identity conflict or regret predicts distress and burnout (Meca et al., 2020; Piotrowski et al., 2023). Older women without children often report both satisfaction and fulfilment, but also regret, depression, or a sense of disrupted identity, reflecting mixed outcomes of childlessness across voluntary and involuntary pathways (Gouni et al., 2022; Graham et al., 2013). Together, these findings illustrate that parental identity functions as a critical psychological construct shaped by choice, constraint, and social context. Extending beyond human parenting, Volsche et al. (2023) found that non-parents and prospective parents often engage in pet caregiving behaviours that mirror human caregiving. This suggests that alternative caregiving roles, such as dog ownership, may fulfil identity needs and contribute to well-being.

Subjective well-being (SWB), typically assessed through life satisfaction and flourishing, is a widely studied indicator of psychological health and adjustment (Diener et al., 1985; Diener et al., 2010). It is conceptualised as a multidimensional construct encompassing cognitive–evaluative elements, such as life satisfaction, and eudaimonic dimensions, including psychological flourishing, meaning, and purpose (Diener et al., 1985). Consistent with this view, research suggests that adults’ SWB is closely tied to the psychological demands and rewards of caregiving roles (Nomaguchi & Milkie, 2020). Identity theory posits that clarity and commitment to salient roles foster emotional resilience, life satisfaction, and flourishing. In a longitudinal study, Fadjukoff et al. (2016) found that adults with strong parental identity reported higher well-being than those with unresolved or diffused identities. Similarly, Piotrowski (2021) demonstrated that strong parental identity commitment was associated with elevated life satisfaction and psychological strengths. Complementing these findings, Piotrowski (2023) showed that low parental identity commitment and heightened reconsideration predicted greater psychological distress and lower well-being. Together, these findings underscore that SWB can be shaped by the degree to which caregiving experiences are psychologically integrated into one’s self-concept, positioning this as a critical outcome for examining the psychological implications of parental identity processes.

The human–pet bond can offer structure, affection, and emotional reciprocity that mirror aspects of parenthood (Barina-Silvestri et al., 2024; Kubinyi, 2025). Daily rhythms of feeding, comforting, and caring for a pet can reinforce role engagement through regular interaction. Owens and Grauerholz (2019) and Volsche (2018) found that women without children often construct pet caregiving as a meaningful life role, using parental language and responsibilities to express their sense of self. Consistent with this perspective, Barina-Silvestri et al. (2024) linked caregiving style to owner and pet well-being, while Volsche (2019) describes this phenomenon as cross-species parenting, suggesting that it may buffer identity-related distress or enhance role fulfilment. Although all companion animals can presumably be ‘parented’, a great deal of existing anthrozoological research has focused on dogs. Evidence indicates that attachment patterns differ across species, with individuals reporting stronger attachment to dogs than cats (Sandøe et al., 2023). This pattern is particularly pronounced among women (Egaña-Marcos et al., 2025). This positions the human–dog bond as a potential moderator of the relationship between parental identity and SWB, in that this bond may alter the strength or direction of identity’s psychological impact. Findings from Allen and Hogg (2022) and Northrope et al. (2024) support the conceptual basis of this framework, demonstrating that the emotional quality of human–dog bonds can interact with individual psychosocial vulnerabilities to influence well-being.

As dogs can become caregiving figures akin to children, it is important to understand their impact on parental identity and SWB. Little research has examined how parental identity and caregiving relationships with companion animals jointly relate to SWB among older women. Research on parental identity has focused primarily on active parents, examining identity development and its links with well-being, without extending this framework to cross-species caregiving relationships (Fadjukoff et al., 2016; Piotrowski, 2021). Conversely, research on the human–dog bond has examined associations with owner well-being and mental health (Merkouri et al., 2022) but has not typically considered parental identity as a psychological framework through which these associations may operate. In parallel, research on pet parenting has described the caregiving roles and meanings people attach to companion animals (Laurent-Simpson, 2017; Volsche, 2019) but has not examined these relationships in relation to SWB within an identity framework. Together, these lines of research suggest that the intersection of parental identity, cross-species caregiving, and SWB may be important, but it remains underexplored, particularly among older women.

Building on this, the present study aimed to test a psychological moderation model examining parental identity, the human–dog bond, and SWB. Women aged 40 and over were chosen as participants, as they represent a key demographic for studying identity renegotiation following reproductive decision-making and important psychosocial transitions (Piotrowski, 2021; Volsche et al., 2023). We also focused on dog owners to enable examination of caregiving-related identity processes in non-parenting contexts, where emotionally meaningful routines and social connection have been shown to enhance well-being in older adults (Barina-Silvestri et al., 2024; Imamatsu et al., 2023). It was hypothesised that parental identity would be positively associated with SWB and that the quality of the dog–human bond would moderate any relationship between these two constructs, such that stronger bonding may either strengthen or buffer the effect of parental identity on SWB.

## 2. Materials and Methods

A total of 296 women aged 40 years and older (*M* = 51.6, *SD* = 8.79, range = 40–78) were recruited via Prolific Academic Ltd., (London, UK) an online recruitment platform that provides access to diverse but non-probabilistic samples across fifty-five countries (https://www.prolific.com; first accessed on 12 November 2025). Eligibility criteria included identifying as female, being aged 40 years or older, owning at least one dog, residing in a Western country (e.g., Australia, New Zealand, Ireland, United States, United Kingdom), and having fluent English proficiency. Women were exclusively included because caregiving roles remain strongly gendered, with more women engaging as primary caregivers within families (Craig & Jenkins, 2016), including those without children (Pacheco Barzallo et al., 2024), than men. Women also often report stronger attachment to companion animals (Laurent-Simpson, 2017). Focusing on women, therefore, allowed the study to examine parental identity and human–animal relationships within a group for whom caregiving is particularly salient. An initial recruitment drive attracted mostly parents (25 June–17 July 2025), so a second round of recruitment was conducted (21 November 2025), limited to non-parents.

An a priori power analysis conducted using G*Power (version 3.1) indicated that 395 participants would be required to detect a small interaction effect (*f*^2^ = 0.02) with *α* = 0.05 and power = 0.80. The final sample did not meet this target and was therefore underpowered to detect small interaction effects; however, this sample size is consistent with those used in comparable moderation studies (Hardie et al., 2023) and provides sufficient power to detect medium effects (Tabachnick & Fidell, 2014).

### 2.1. Demographic Variables

A number of socioeconomic variables were assessed, including income and education, which are consistently linked to well-being outcomes, with higher income and socioeconomic status associated with better mental health and life satisfaction (Navarro-Carrillo et al., 2020; Thomson et al., 2022), and education contributing to well-being through its influence on health behaviours and long-term life outcomes (Olsen et al., 2023). Relationship status was also measured as a covariate, given evidence that individuals in stable romantic relationships tend to report higher psychological well-being than those who are single (Sagone et al., 2023). Age was assessed as a standard demographic factor known to relate to changes in health and well-being across the lifespan (Plácido et al., 2022).

### 2.2. Parental Identity

Parental identity was assessed using two measures. First, participants indicated whether they had ever identified as a parent to one or more human children (yes/no). Parents then rated the voluntariness of becoming a parent on a 100-point scale (0 = completely involuntary, 100 = completely voluntary), as well as the strength of their parental identity and the extent of time devoted to the parenting role using 7-point Likert scales. Non-parents rated the voluntariness of not becoming a parent using the same 100-point scale.

The second measure was a 21-item Parental Identity and Enjoyment Scale (PIES), designed specifically for this project to capture the complex and individualised psychological experience of parenting and non-parenting across all adults, including those who are voluntarily or involuntarily childfree or childless. There are limited existing measures of parental identity, particularly those that are inclusive of diverse parenting experiences. One of the few available tools is the Utrecht-Management of Identity Commitments Scale (U-MICS), which was adapted from a general identity framework to assess parental identity development (Piotrowski, 2018). Because this adaptation was developed specifically for use with parental populations, it excludes individuals whose identities form in the absence of children. It was therefore modified for the current study with the ENJOY Scale, developed by Davidson et al. (2023), and used to measure universal, multidimensional enjoyment, also being used to inform the tone, affective emphasis, and focus on domain-specific fulfilment in the development of the new measure. Based on responses to the initial parental status measure, participants completed a parenting- or non-parenting-framed version of the PIES, with each item being rated on a 7-point Likert scale (1 = Strongly disagree, 7 = Strongly agree). Example items include “I enjoy being a parent” (parent version) and “I enjoy not being a parent” (non-parent version). Exploratory factor analysis was performed on the PIES, following conventions outlined by Costello and Osborne (2005) to reveal any latent variables, but no satisfactory solution could be obtained, with cross-loadings and high factor intercorrelations leading to a single factor being retained. Hence, parental identity orientation was assessed using the mean PIES score, calculated by summing participants’ responses to all 21 items and dividing by the total number of items, resulting in a composite score ranging from 1 to 7. Full item sets are provided in the Supplementary Materials.

### 2.3. Subjective Well-Being

SWB was assessed using two validated measures. The Satisfaction With Life Scale (SWLS) measured global life satisfaction using five items rated on 7-point Likert scales (1 = strongly disagree to 7 = strongly agree; Diener et al., 1985). Higher scores reflect greater overall satisfaction with one’s life. An example item is “I am satisfied with my life”. The Flourishing Scale (FS) measured psychological flourishing, including purpose, competence, and social connectedness, using eight items rated on the same scale (Diener et al., 2010). Higher total scores indicate stronger psychological functioning and more positive self-evaluations across these domains. An example item is “I am optimistic about my future”. The SWLS has demonstrated good internal consistency across samples, with Cronbach’s α typically ranging from 0.79 to 0.90 (Hardie et al., 2023; Tong & Wang, 2017). The FS has similarly shown high internal reliability, with Cronbach’s α values commonly reported between 0.88 and 0.92 (Espejo et al., 2022; Tong & Wang, 2017). Together, these measures capture complementary cognitive and psychological dimensions of SWB. Koreki et al. (2022) and Kosugi et al. (2021) previously validated the SWLS alongside the FS in Japanese populations, supporting the integration of both scales into comprehensive assessments of SWB.

### 2.4. Human–Dog Bond

The Dog Owner Relationship Scale (DORS-28) was used to assess the quality of participants’ relationships with their dogs (Bennett et al., 2025). The scale consists of 28 items across four subscales: Perceived Costs, Affectionate Engagement, Emotional Reliance, and Active Engagement. Items are rated on 7-point scales, with higher total scores indicating stronger human–dog bonds. The total DORS-28 score was used as a continuous moderator in regression analyses. The DORS-28 has demonstrated excellent internal consistency, with a Cronbach’s α of 0.92 (Bennett et al., 2025). An example item is “My dog is constantly attentive to me.” All DORS-28 items are provided in the Supplementary Materials.

Participants completed a brief screening survey via Prolific to assess eligibility, followed by the main survey hosted on REDCap (https://project-redcap.org). After providing electronic informed consent, participants completed a fixed-sequence questionnaire assessing demographic questions, parental identity, SWB and the human–dog relationship. The survey took approximately 8–10 min to complete. Participants were compensated in accordance with Prolific’s fair payment guidelines at an estimated rate of £6.00 per hour.

### 2.5. Statistical Analysis

This study employed a cross-sectional survey design. Data were analysed using SPSS (version 29). Prior to hypothesis testing, data screening and assumption checks were conducted including inspection of missing data, normality, and linearity. Descriptive statistics and internal consistency estimates were calculated for all study variables. Pearson correlations were conducted to examine associations between parental identity and SWB. Moderated multiple regression analyses were then conducted to test whether the human–dog relationship moderated associations between parental identity and well-being outcomes. Variables were mean-centred prior to computing interaction terms. Where significant interactions were observed, conditional effects were examined at low (−1 SD), mean, and high (+1 SD) levels of the moderator. All analyses used an alpha level of 0.05. Sociodemographic variables were included as covariates in the models, selected based on their established associations with SWB. Including these covariates allowed for a more precise estimation of the unique contributions of parental identity, voluntariness, and the human–dog bond to SWB. Bootstrapped estimates (5000 resamples) were examined for all moderation models, with results remaining consistent and showing no meaningful differences from the original models.

## 3. Results

### 3.1. Sample Characteristics

A total of 296 women participated in this study, with ages ranging from 40 to 78 years (*M* = 51.6, *SD* = 8.8). Analysis of the demographic information (Table 1) revealed that although most participants were parents (*n* = 177, 58.8%), 122 women (41.2%) reported being non-parents. Participants primarily resided in the United States, the United Kingdom, Australia and Canada. Educational attainment was generally high, with 40.5% of participants holding a university degree and 20.6% reporting postgraduate qualifications. An additional 23.6% had completed vocational training. Work status varied across the sample. More than half (56.8%) were employed full-time, while 23.6% reported part-time employment. Smaller proportions identified as retired (9.1%), engaged in home duties (5.7%), or falling into other categories (8.7%). Participants were permitted to endorse more than one work status category, and 12 participants (4.1%) selected multiple options. Overall, the sample reflected a diverse but predominantly well-educated and employed group of older women. In terms of relationship status, 42.6% of participants were married, with a further 22.0% separated, divorced, or widowed.

All participants were dog owners, with most owning a single dog (73.6%), while 20.9% owned two dogs, and only a small number owned three or more (5.5%). Most participants had long-term experience with dogs, with 80.1% reporting ownership of more than three years, 15.9% between one and three years, and only 4.1% less than one year.

### 3.2. Descriptive Statistics for Key Variables

Internal consistency was high for all scales, with Cronbach’s α values (Table 2) indicating excellent reliability (Pallant, 2020). Participants reported relatively high parental identity (*M* = 5.94, *SD* = 0.75) and strong dog–owner relationship scores (*M* = 5.55, *SD* = 0.79), alongside moderately high levels of life satisfaction (*M* = 4.47, *SD* = 0.41) and psychological flourishing (*M* = 5.54, *SD* = 0.30).

### 3.3. Parental Status and Voluntariness

Among participants, 174 identified as parents and 122 as non-parents. For the parents, ratings of parental voluntariness were generally high (*M* = 87.07, *SD* = 23.55, range = 0–100). In contrast, non-parents reported lower and more variable voluntariness scores (*M* = 73.61, *SD* = 29.49, range = 0–100).

### 3.4. Parental Identity and Subjective Well-Being

A significant positive association was found between parental identity and life satisfaction, *r*(294) = 0.38, *p* < 0.001, and between parental identity and psychological flourishing, *r*(294) = 0.23, *p* < 0.001. Based on Cohen’s (2013) guidelines, the association between parental identity and life satisfaction represents a moderate effect, while the association with flourishing represents a small-to-moderate effect. Hence, higher parental identity was meaningfully associated with greater life satisfaction and psychological flourishing.

### 3.5. Main Moderation Analyses

**Figure 1:**
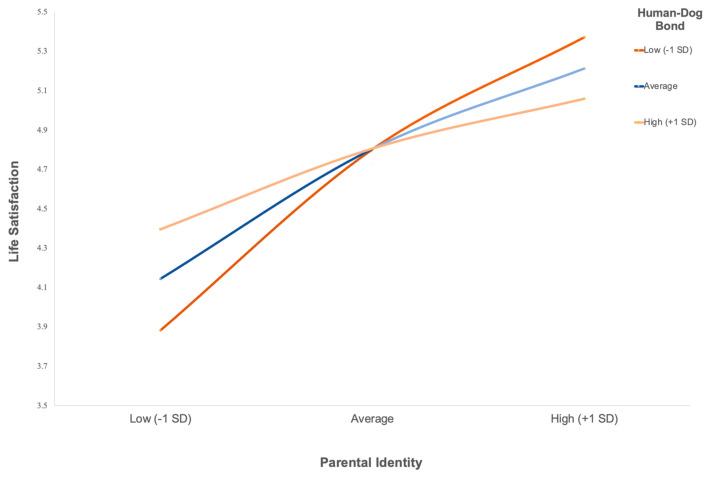
*Parental Identity (PIES) × Human–Dog Bond (DORS-28) Predicting Life Satisfaction (SWLS).* Note. Predicted values are plotted at low (−1 *SD*), mean, and high (+1 *SD*) levels of parental identity (PIES) and the human–dog bond (DORS-28), estimated from moderated regression (PROCESS) with mean-centred variables.

Two moderated regression analyses were conducted to test whether the human–dog bond moderated the relationship between parental identity and SWB. For the first model, with life satisfaction as the outcome, the overall regression model was significant, *R*^2^ = 0.151, *F*(3, 292) = 17.33, *p* < 0.001. As shown in Table 3, parental identity was a significant positive predictor of life satisfaction (*b* = 0.518, *SE* = 0.072, *p* < 0.001), whereas the human–dog bond was not a significant predictor (*b* = −0.002, *SE* = 0.036, *p* = 0.946). The interaction between parental identity and the human–dog bond was not significant (*b* = −0.037, *SE* = 0.032, *p* = 0.251) and did not account for a significant increase in explained variance (Δ*R*^2^ = 0.004, *F*(1, 292) = 1.33, *p* = 0.251). Table 4 presents the conditional effects of parental identity on life satisfaction at low, mean, and high levels of the human–dog bond. Figure 1 was examined to aid interpretation of the conditional effects, confirming that parental identity was positively associated with life satisfaction across all levels of the human–dog bond.

**Figure 2:**
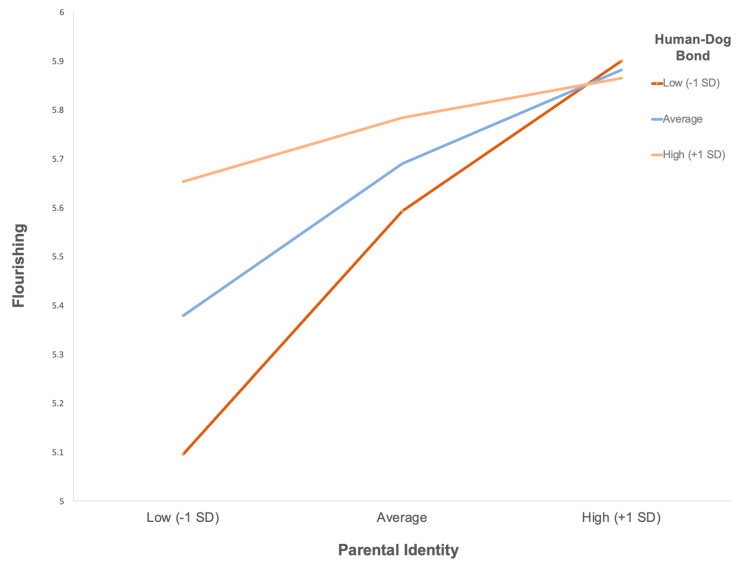
*Parental Identity (PIES) × Human–Dog Bond (DORS-28) Predicting Flourishing (FS).* Note. Predicted values are plotted at low (–1 *SD*), mean, and high (+1 *SD*) levels of parental identity (PIES) and the human–dog bond (DORS-28), estimated from moderated regression (PROCESS) with mean-centred variables.

For the second model, with psychological flourishing as the outcome, the overall regression model was significant, *R*^2^ = 0.067, *F*(3, 292) = 6.99, *p* < 0.001. As shown in Table 3, parental identity was a significant positive predictor of flourishing (*b* = 0.208, *SE* = 0.048, *p* < 0.001), while the human–dog bond was not a significant predictor (*b* = 0.040, *SE* = 0.024, *p* = 0.096). The interaction between parental identity and the human–dog bond was not significant (*b* = −0.031, *SE* = 0.021, *p* = 0.151), with no significant change in explained variance (Δ*R*^2^ = 0.007, *F*(1, 292) = 2.07, *p* = 0.151). Table 4 presents the conditional effects of parental identity on flourishing at low, mean, and high levels of the human–dog bond. Figure 2 was used to aid interpretation of the conditional effects, indicating that parental identity was positively associated with flourishing across all levels of the human–dog bond.

### 3.6. Secondary Moderation Analyses

Two additional moderated regression analyses were conducted, replacing parental identity with voluntariness and including a number of potential covariates. For the model predicting life satisfaction, the overall regression model was statistically significant, *R*^2^ = 0.191, *F*(8, 287) = 8.47, *p* < 0.001. As shown in Table 5, voluntariness was not a significant predictor of life satisfaction (*b* = 0.005, *p* = 0.091), and the human–dog bond was also not a significant predictor (*b* = 0.032, *p* = 0.378). The interaction between voluntariness and the human–dog bond did not reach statistical significance (*b* = −0.003, *p* = 0.062), indicating that the association between voluntariness and life satisfaction did not vary as a function of the human–dog bond. Among the covariates, income emerged as a significant positive predictor (b=0.692, p<0.001), and parental status was also significant, with parents reporting higher life satisfaction than non-parents (*b* = 0.385, *p* = 0.041), whereas age, education, and relationship status were not significant predictors. Conditional effects analyses (see Table 6 and Supplementary Materials) indicated that voluntariness did not significantly predict life satisfaction at low, mean, or high levels of the human–dog bond.

For the model predicting flourishing, the overall regression model was statistically significant, *R*^2^ = 0.154, *F*(8, 287) = 6.54, *p* < 0.001. Voluntariness was not a significant predictor of flourishing (*b* = 0.000, *p* = 0.933), but the human–dog bond emerged as a significant positive predictor (*b* = 0.057, *p* = 0.017). The interaction between voluntariness and the human–dog bond was statistically significant (*b* = −0.002, 95%CI [−0.004, −0.000], *p* = 0.025), indicating that the association between voluntariness and flourishing varied as a function of the human–dog bond. However, conditional effects analyses (see Table 6) indicated that voluntariness did not significantly predict flourishing at low, mean, or high levels of the human–dog bond. Parental status did not emerge as a significant covariate (b=0.179, p=0.141), whereas income was a significant positive predictor (b=0.377, p<0.001). Age approached significance (b=0.012, p=0.068), while education and relationship status were not significant predictors.

## 4. Discussion

The aim of this study was to examine whether parental identity is associated with subjective well-being (SWB) in older women who own dogs, and to test whether the strength of the human–dog bond moderates this relationship. Based on previous research, it was expected that parental identity would be positively associated with SWB, and that the human–dog bond would moderate this association, such that stronger bonds would either buffer weaker parental identity or amplify stronger identity. Parental identity was positively associated with both life satisfaction and flourishing, indicating that older women who reported stronger identification with the parenting role also reported higher levels of evaluative and eudaimonic well-being. This is consistent with prior research demonstrating that parental identity salience and coherence are associated with greater life satisfaction and psychological functioning (Espejo et al., 2022; Koreki et al., 2022; Kosugi et al., 2021; Tong & Wang, 2017). It extends this previous research by confirming that these associations are observed even within a sample that included a relatively high proportion of non-parents. This strengthens the interpretation of parental identity as a distinct psychological construct rather than simply reflecting parenting status; internalised caregiving identities remain psychologically meaningful even in the absence of enacted parenting roles.

Evidence for the second hypothesis was more limited than expected. The human–dog bond did not moderate the association between parental identity and either life satisfaction or psychological flourishing. For life satisfaction, the interaction between parental identity and the human–dog bond was not statistically significant, indicating that the association between parental identity and evaluative well-being did not vary as a function of bond strength. Instead, parental identity independently predicted higher life satisfaction and conditional effects analyses demonstrated that this association was present at low, mean, and high levels of the human–dog bond. Emotional closeness to a dog, therefore, did not compensate for weaker parental identity in shaping global life evaluations. A similar pattern was observed for psychological flourishing. Although parental identity was positively associated with flourishing across all levels of the human–dog bond, the interaction term was not statistically significant, indicating that the strength of this association did not differ meaningfully across levels of emotional closeness to a dog. The significant conditional effects reflect a stable main effect of parental identity rather than a moderating process. Together, these findings suggest that the human–dog bond may not function as a compensatory mechanism for lower parental identity but may instead operate as a broader relational context within which identity–well-being associations occur. Consistent with theoretical perspectives on cross-species caregiving, human–pet relationships may be identity-relevant and psychologically meaningful without serving as direct substitutes for human parenting roles (Volsche, 2018, 2019; Volsche et al., 2023).

The secondary analyses examining voluntariness provided additional insight into how different dimensions of parental orientation relate to well-being. Voluntariness was not associated with life satisfaction, nor did the human–dog bond alter this relationship, indicating that perceived choice regarding parenthood was not a strong predictor of evaluative well-being in this sample. Instead, parental status itself appeared to be more closely linked to life satisfaction, suggesting that global life evaluations may be shaped more strongly by life circumstances than by subjective appraisals of choice. Covariate patterns also suggest that broader structural factors may play a central role. Income emerged as a consistent and robust positive predictor of life satisfaction, while parental status also showed a significant association, indicating that global life evaluations may be shaped more strongly by objective life circumstances and resources than by subjective appraisals of choice.

A more complex pattern emerged for psychological flourishing. Although voluntariness was not directly associated with flourishing at low, mean, or high levels of the human–dog bond, the presence of a statistically significant interaction suggests that the relationship between voluntariness and eudaimonic well-being may be influenced by broader relational contexts. However, because this interaction did not translate into clearly differentiated conditional effects, it does not support a straightforward moderating or buffering interpretation. Rather, the findings point to a subtle interplay between perceived parental choice and the human–dog bond that does not manifest as distinct differences in flourishing across levels of the moderator. Importantly, the covariate structure differed from that observed for life satisfaction. Income again emerged as a strong positive predictor, highlighting the role of material resources in supporting broader functioning and life engagement. In contrast, parental status did not significantly predict flourishing, suggesting that eudaimonic well-being may be less dependent on categorical life roles and more reflective of ongoing psychological and contextual resources. These findings reinforce the distinction between evaluative and eudaimonic well-being, with life satisfaction appearing more sensitive to structural life circumstances, and flourishing reflecting a more complex integration of relational and personal factors.

Taken together, the findings support two key conclusions. First, parental identity appears to be a more psychologically meaningful predictor of SWB in older women than either parental status or perceived voluntariness of parental status. Notably, across models, income consistently emerged as a significant predictor of well-being, underscoring the importance of material resources in shaping both evaluative and eudaimonic outcomes, whereas parental status was only associated with life satisfaction. Second, the human–dog bond plays a limited and context-dependent role in shaping well-being, particularly in relation to flourishing. By including a sample of parents and non-parents, the present study clarifies that, while companion animals may contribute to SWB, they do so in ways that complement rather than replace the well-being benefits associated with strong parental identity.

These findings have several implications for theory, research, and practice. While prior research has predominantly conceptualised parental identity within the context of active childrearing (Piotrowski, 2021; Schrooyen et al., 2021), the present findings indicate that identity salience and enjoyment are associated with both life satisfaction and flourishing even in a sample that included a more balanced distribution of parents and non-parents. This reinforces the view that parental identity reflects an internalised domain of self-concept rather than a simple by-product of demographic status. The absence of consistent effects for voluntariness further supports this interpretation, suggesting that the psychological significance of parental identity lies in the strength and coherence of identification with the parenting role, rather than in whether one’s parental or non-parental pathway is perceived as voluntary. It should be acknowledged, nonetheless, that the magnitude of the effects was modest. This is consistent with prior identity and well-being research (Piotrowski, 2021; Schrooyen et al., 2021) and likely reflects the multifactorial nature of SWB. At present, parental identity can be understood as one psychological factor associated with, rather than determining, well-being outcomes. Future studies may benefit from incorporating additional psychological variables, such as meaning in life, attachment style, or role conflict, to better capture the complexity of these processes. Research designs with more participants and, therefore, more power to detect small effects, are also recommended. Increasing the power of the study would permit inclusion of potentially important sociodemographic and contextual confounders. Although we included some variables as covariates in our analyses, many other factors may plausibly influence parental identity, the strength of the human–dog bond, and SWB, meaning that the observed associations may be partially attributable to these unmeasured influences. Future research should address this by incorporating preregistered covariate plans, applying consistent and standardised measurement approaches, and considering sampling strategies that reduce heterogeneity and improve causal interpretability.

From an anthrozoological perspective, the findings contribute to a more nuanced understanding of the role of the human–dog bond in women’s well-being. Although the human–dog bond did not uniformly moderate the association between parental identity and SWB, the analyses suggest that the bond may be more relevant for certain aspects of well-being, particularly flourishing. Importantly, conditional effects analyses suggested that the human–dog bond does not operate as a straightforward substitute for parental identity, but rather as an additional relational context that may support psychological functioning in more diffuse or structural ways. This pattern is consistent with broader critiques in human–animal interaction research, which emphasise substantial variability in how and for whom relationships with companion animals influence well-being, as well as outcome-specific and context-dependent effects (Rodriguez et al., 2021). By highlighting outcome-specific effects, the present findings refine existing anthrozoological models and caution against overly simplistic claims that strong bonds with animals compensate for unmet parenting identities.

For mental health professionals working with older women navigating identity-related transitions, including involuntary childlessness, delayed parenthood, or caregiving role changes, the findings underscore the importance of recognising parental identity as a salient psychological domain regardless of parental status. The human–dog bond may represent a meaningful source of emotional closeness, routine, and relational engagement, particularly in relation to flourishing, but should not be assumed to fully offset challenges associated with weaker parental identity. While not the focus of the present study, this suggests clinical value in exploring clients’ relationships with companion animals as part of a broader assessment of identity and well-being, while remaining attentive to the limits of such bonds.

A strength of this study was the use of a sample drawn from Prolific, which resulted in the inclusion of a relatively broad sample of dog-owning Western women, rather than a highly self-selected group of individuals strongly identifying as “pet parents”. Very different results might have been obtained had the sample been drawn solely from those who feel very close to their dog or who describe themselves as ‘pet parents’. The focus on women in Western cultural contexts was strategic, as prior research suggests that such women are likely to report strong emotional engagement with companion animals and to adopt caregiving roles toward pets, making this population particularly relevant for examining identity-related caregiving processes during a key developmental stage (Fadjukoff et al., 2016; Laurent-Simpson, 2017). The focus on dogs was also strategic, as evidence indicates that individuals report stronger attachment to dogs than cats (Sandøe et al., 2023), a pattern that is particularly pronounced among women (Egaña-Marcos et al., 2025). Nonetheless, restricting the sample in this way also limits the generalisability of the findings. It will be of interest to examine other groups in future. Men, younger adults, individuals from non-Western cultures and people who own pets other than dogs, may experience parental identity and pet caregiving differently. Even within the key demographic, generalisability is restricted by recruitment using a non-probability sampling approach. Non-probability online samples are subject to self-selection and platform-based recruitment, meaning participants are typically active internet users who proactively choose to take part in research, which may introduce systematic biases in representativeness (Lehdonvirta et al., 2020). To address this, future studies could recruit participants across multiple platforms, use Prolific’s filtering tools to align sample demographics with population distributions reported in national census data or even use traditional offline data capture approaches to ensure representation across multiple groups. Other pet types could also be included. Replication with larger and more diverse samples and incorporation of longitudinal designs would strengthen the robustness and generalisability of future research and improve the power to detect small effects, which was limited in this study. Examining potential mediating processes, including perceived meaning, daily structure, or emotional regulation, may also clarify how and when the human–dog bond contributes to psychological functioning in specific samples.

It will also be important in future work to validate the measure of parental identity created for this study. The Parental Identity and Enjoyment Scale (PIES) demonstrated strong internal consistency and conceptual coherence and provides a novel, theoretically grounded, tool capable of capturing parental identity beyond categorical parenting status. Nevertheless, further psychometric evaluation is required, including factor analyses in a larger and more diverse sample and cross-cultural measurement testing. The use of novel instruments is necessary when no other options are available but necessarily reduces confidence in any conclusions drawn.

## 5. Conclusions

The findings of this study suggest that parental identity is a more psychologically meaningful predictor of subjective well-being in older women than either parental status or perceived voluntariness of parental status. They also suggest that, in a broad sample of dog-owning Western women aged over 40 years who voluntarily participate in online research, the human–dog bond plays a limited but potentially important role in shaping well-being, particularly in relation to flourishing. Secondary analyses indicated that income was a consistent positive predictor of well-being, while parental status was associated with life satisfaction but not flourishing. These findings highlight the value of examining caregiving relationships beyond traditional parenting contexts when considering factors associated with well-being, suggesting that internalised caregiving identities may contribute to well-being independently of parental status. We therefore suggest that recognising the diversity of caregiving identities, including those expressed through the human–dog bond, may help inform more inclusive perspectives on identity and well-being in older women. While companion animals may contribute to subjective well-being, they appear to do so in ways that complement rather than replace the psychological benefits associated with strong parental identity. Given the modest magnitude of the effects, the specificity of the sample, and the limited power of the study to detect small effects or control adequately for potential confounders, these conclusions should be interpreted with caution and may not generalise beyond the current sample.

## Figures and Tables

**Table 1:** Sample Demographics for *N* = 296 women.

Demographics	*n*	%
Age (years)		
40–49	137	46.3
50–59	112	35.5
60–69	42	13.7
70–79	12	4.5
Parental status		
Parent	174	58.8
Non-parent	122	41.2
Residence		
Australia	22	7.4
New Zealand	2	0.7
United States	176	59.5
Canada	23	7.8
United Kingdom	73	24.7
Ireland/Other	0	0
Education		
No formal schooling/Year 10 or below	7	2.4
High school	36	12.2
Vocational/TAFE	70	23.6
University	120	40.5
Postgraduate	61	20.6
Other	2	0.7
Work status		
Full-time	168	56.8
Part-time	70	23.6
Retired	27	9.1
Home duties	17	5.7
Other	26	8.7
Relationship status		
Single/Never married	67	22.6
In a relationship (not living together)	14	4.7
De facto/common law or living together	23	7.8
Married	126	42.6
Separated/Divorced/Widowed	65	22
Prefer not to say	1	0.3
Household adults		
1	94	31.8
2	124	41.9
3	54	18.2
4 or more	24	8.1
Household children		
0	209	70.6
1	49	16.6
2	24	8.1
3	9	3.0
4 or more	5	1.7
Children’s age categories		
0–4 years	9	3.0
5–8 years	26	8.8
9–12 years	32	10.8
13–17 years	58	19.6
Number of dogs		
1	218	73.6
2	62	20.9
3	10	3.4
4	4	1.4
6 or more	2	0.7
Dog ownership duration		
Less than 1 year	12	4.1
1–3 years	47	15.9
More than 3 years	237	80.1

Note. Participants could select more than one work status category; percentages therefore exceed 100%.

**Table 2:** Mean (*M*), Standard Deviation (*SD*), Range, and Reliability (α) for all Measures.

Scale	*M*	*SD*	Possible Range	Observed Range	Cronbach’s α
Parental Identity and Enjoyment Scale (PIES)	5.94	0.75	1–7	1–7	0.90
Dog Owner Relationship Scale (DORS-28)	5.55	0.79	1–7	9–21	0.91
Satisfaction With Life Scale (SWLS)	4.47	0.41	1–7	1–7	0.93
Flourishing Scale (FS)	5.54	0.30	1–7	1–7	0.91

**Table 3:** Multiple Regression of Parental Identity (PIES), Human–Dog Bond (DORS-28), and their Interaction Predicting Life Satisfaction (SWLS) and Flourishing (FS).

			95%CI	
Predictors	*B*	*SE*	LL	UL
SWLS				
PIES	0.518	0.07	0.38	0.66
DORS-28	−0.002	0.04	−0.10	0.03
PIES × DORS-28	−0.037	0.03	−0.10	0.03
FS				
PIES	0.21	0.05	0.11	0.30
DORS-28	0.04	0.02	−0.01	0.09
PI × DORS-28	−0.03	0.02	−0.07	0.01

Note. CI = confidence interval; LL = lower limit; UL = upper limit.

**Table 4:** Simple Slopes Analyses of Parental Identity (PIES) Predicting Well-Being at Low, Mean, and High Levels of the Human–Dog Bond (DORS-28).

					95%CI	
Predictors	*B*	*SE*	*t*_(292)_	*p*	LL	UL
SWLS						
Low (−1 SD; −2.299)	0.603	0.116	5.18	0.001 ***	0.374	0.833
Mean (2.299)	0.518	0.072	7.16	0.001 ***	0.375	0.660
High (+1 SD; +2.299)	0.432	0.089	4.83	0.001 ***	0.256	0.608
FS						
Low (−1 SD; −2.299)	0.279	0.077	3.61	0.001 ***	0.127	0.431
Mean (2.299)	0.208	0.048	4.34	0.001 ***	0.113	0.302
High (+1 SD; +2.299)	0.137	0.059	2.31	0.022 *	0.020	0.253

Note. CI = confidence interval; LL = lower limit; UL = upper limit. * *p* < 0.05. *** *p* < 0.001.

**Table 5:** Multiple Regression of Voluntariness, Human–Dog Bond (DORS-28), and their Interaction Predicting Life Satisfaction (SWLS) and Flourishing (FS).

			95%CI	
Predictors	*B*	*SE*	LL	UL
SWLS				
Voluntariness	0.005	0.003	−0.001	0.012
DORS-28	0.032	0.037	−0.040	0.104
Voluntariness × DORS-28	−0.003	0.001	−0.006	0.000
Parental Status	0.385	0.188	0.016	0.755
Age	0.003	0.010	−0.17	0.22
Education	0.54	0.080	−0.104	0.22
Income	0.692	0.105	0.486	0.899
Relationship Status	0.077	0.62	−0.45	0.200
FS				
Voluntariness	0.000	0.002	−0.004	0.004
DORS-28	0.057	0.024	−0.010	0.104
Voluntariness × DORS-28	−0.002	0.001 ***	−0.004	0.000
Parental Status	0.179	0.121	−0.060	0.418
Age	0.012	0.006	−0.001	0.24
Education	0.072	0.052	−0.031	0.174
Income	0.377	0.068	0.244	0.511
Relationship Status	−0.003	0.040	−0.082	0.76

Note. CI = confidence interval; LL = lower limit; UL = upper limit. *** *p* < 0.001.

**Table 6:** Simple Slopes Analyses of Voluntariness Predicting Life Satisfaction (SWLS) and Flourishing (FS), at Low, Mean, and High Levels of the Human–Dog Bond (DORS-28).

					95%CI	
*Predictor: Voluntariness*	*B*	*SE*	*t*_(200)_	*p*	LL	UL
SWLS						
Low (−1 SD; −2.507)	0.0012	0.005	2.55	0.011	0.003	0.022
Mean (2.571)	0.005	0.003	1.45	0.145	−0.002	0.011
High (+1 SD; +2.337)	−0.001	0.005	−0.217	0.828	−0.010	0.008
FS						
Low (−1 SD; −2.507)	0.005	0.003	1.77	0.078	−0.001	0.012
Mean (2.507)	0.000	0.002	−0.200	0.985	−0.004	0.004
High (+1 SD; +2.507)	−0.005	0.003	−1.578	0.116	−0.011	0.001

Note. CI = confidence interval; LL = lower limit; UL = upper limit.

## Data Availability

Due to ethical restrictions, the data can only be made available upon request to the authors and approval from the relevant Ethics Committee.

## Supplementary Materials

The following supporting information can be downloaded at: https://www.mdpi.com/article/10.3390/bs16040567/s1, S1: Participant Questionnaire; S2: Simple Slopes Figures for Secondary Moderation Models; Figure S1: Voluntariness × Human–Dog Bond (DORS-28) Predicting Life Satisfaction (SWLS); Figure S2: Voluntariness × Human–Dog Bond (DORS-28) Predicting Flourishing (FS).

## Abbreviations

The following abbreviations are used in this manuscript:

SWB	Subjective well-being
SWLS	Satisfaction With Life Scale
FS	Flourishing Scale
PIES	Parental Identity and Enjoyment Scale
DORS-28	Dog Owner Relationship Scale (28-item version)
