# Supplementary material for: Parental Identity and Subjective Well-Being in Older Women: The Moderating Role of the Human–Dog Bond
Source: Behav Sci (Basel). 2026 Apr 9;16(4):567. doi: 10.3390/bs16040567 (PMC13113525; doi:10.3390/bs16040567)
Supplement: Supplementary file 1 [file behavsci-16-00567-s001.zip › behavsci-4154433-supplementary.pdf]

Supplementary Materials

S1: Participant Questionnaire

What is your Prolific ID?

Do you own a dog that lives with you?

☐ Yes

☐ No [exit survey]

How many dogs do you own who live with you?

☐ 1

☐ 2

☐ 3

☐ 4

☐ 5

☐ 6 or more

What is your gender?

(Please select one)

☐ Man

☐ Woman

☐ Non-binary / third gender

☐ Prefer not to say

☐ I identify my gender as...

Please write_________________________

In what year were you born?

(Drop Down List)

☐ 1925

☐ 1985

Where do you currently reside?

☐ Australia

☐ New Zealand

☐ United States

☐ Canada

☐ United Kingdom

☐ Ireland

☐ Other

Please write___________________

What is the highest level of education you have completed?

(Please select one)

☐ No formal schooling
☐ Year/Grade 10 or below (up to age 16 years)
☐ Year/Grade 11 or 12 (above age 16 years)
☐ Certificate, diploma, advanced diploma, associate degree, technical/trade qualification, TAFE
☐ University/College degree (Bachelor's degree)
☐ University/College degree (Master's, PhD, or equivalent)
☐ Prefer not to say
☐ Other
Please write__________________

Which of the following best describes your current situation in relation to paid work?
☐ Retired
☐ Unemployed
☐ Unable to work
☐ Engaged in home duties
☐ Part time/casual paid work (30 hours or less per week)
☐ Full time paid work (more than 30 hours per week)
☐ Student
☐ I'd rather not say
☐ Other
Please write__________________

Relative to other people in your community, how would you describe your household income?
☐ Much lower than average
☐ Lower than average
☐ About average
☐ Higher than average
☐ Much higher than average

In total, including yourself, how many adults aged 18 or over live in your household most of the time?
☐ 1
☐ 2
☐ 3
☐ 4 or more

How many children aged 17 years or younger live in your household most of the time?
☐ 0
☐ 1
☐ 2
☐ 3
☐ 4 or more

Please indicate the age(s) of the child/children in your home. Please select all that apply.

☐ 0 to 4 years
☐ 5 to 8 years
☐ 9 to 12 years
☐ 13 to 17 years

Which of these, best describes your current relationship status?
☐ Single/Never married
☐ De-facto/common law or living together
☐ Married
☐ Separated, divorced, or widowed
☐ I'd rather not say

Parental Status & Voluntariness

During your adult life, would you ever have described yourself as being a parent to one or more human children?
☐ Yes
☐ No
[If "Yes", answer the following:]
To what extent did you voluntarily choose to be a parent?
(0 = Completely involuntary, 100 = Completely voluntary)
Indicated on a slider from 0-100

Throughout your adult life, to what degree have you identified with the role of being a parent to a human child or children?
(1 = Not at all, 7 = Very strongly)
☐ 1
☐ 2
☐ 3
☐ 4
☐ 5
☐ 6
☐ 7

Throughout your adult life, how much time have you devoted to the role of being a parent?
(1 = None at all, 7 = A very large amount)
☐ 1
☐ 2
☐ 3
☐ 4
☐ 5
☐ 6
☐ 7

Throughout your adult life, how many children have you been a parent for (biological, adopted, or in a caregiving role)?

Please write_______________

[If "No", answer the following:]

To what extent did you voluntarily choose not to be a parent?
(0 = Completely involuntary, 100 = Completely voluntary)
Indicated on a slider from 0-100

Parental Identity and Enjoyment

Set A Instructions: Please indicate the degree to which you agree with each statement as it relates to your status as a parent (1 = Strongly Disagree, 7 = Strongly Agree)	*Set B Instructions:* Please indicate the degree to which you agree with each statement as it relates to your status as not being a parent. (1 = Strongly Disagree, 7 = Strongly Agree)
**1.** I enjoy being a parent	**1.** I enjoy not being a parent
**2.** Being a parent brings me happiness	**2.** Not being a parent brings me happiness
**3.** I feel proud of being a parent	**3.** I feel proud of not being a parent
**4.** My day-to-day life is enriched by being a parent	**4.** My day-to-day life is enriched by not being a parent
**5.** I find emotional comfort in being a parent	**5.** I find emotional comfort in not being a parent
**6.** Having a child or children is an important part of who I am	**6.** Not having a child or children is an important part of who I am
**7.** Having a child or children defines how I see myself	**7.** Not having a child or children defines how I see myself
**8.** Having a child or children is one of the first things I mention when talking about myself	**8.** Not having a child or children is one of the first things I mention when talking about myself
**9.** Having a child or children reflects who I truly am	**9.** Not having a child or children reflects who I truly am
**10.** My position in relation to having a child or children influences how others see me	**10.** My position in relation to not having a child or children influences how others see me
**11.** I often think about how others judge my decision or situation regarding having children	**11.** I often think about how others judge my decision or situation regarding not having children
**12.** I feel that my parenting status was mostly my choice	**12.** I feel that my non-parenting status was mostly my choice
**13.** I accepted my parenting path willingly	**13.** I accepted my non-parenting path willingly
**14.** I did not feel pressured into the parenting path I'm on.	**14.** I did not feel pressured into the non-parenting path I'm on
**15.** I feel empowered in the role I have, whether or not I have children	**15.** I feel empowered in the role I have, whether or not I have children
**16.** My life turned out as I intended in terms of having children	**16.** My life turned out as I intended in terms of having children
**17.** I regret having a child or children.	**17.** I regret not having a child or children.
**18.** I often reflect on what my life would have been like if I had not had a child or children	**18.** I often reflect on what my life would have been like if I had a child or children

**19.** If I had my time again, I would prefer to not have a child or children	**19.** If I had my time again, I would prefer to have a child or children
**20.** My future would be more secure if I did not have a child or children	**20.** My future would be more secure if I had a child or children
**21.** My life would have been more interesting if I did not have a child or children	**21.** My life would have been more interesting if I had a child or children

Satisfaction with Life Scale (SWLS)

Instructions:

Below are five statements that you may agree or disagree with. Indicate your agreement with each item (1 = Strongly Disagree, 7 = Strongly Agree)

1. In most ways my life is close to my ideal.

☐ 1
☐ 2
☐ 3
☐ 4
☐ 5
☐ 6
☐ 7

2. The conditions of my life are excellent.

☐ 1
☐ 2
☐ 3
☐ 4
☐ 5
☐ 6
☐ 7

3. I am satisfied with my life.

☐ 1
☐ 2
☐ 3
☐ 4
☐ 5
☐ 6
☐ 7

4. So far I have gotten the important things I want in life.

☐ 1
☐ 2
☐ 3
☐ 4
☐ 5
☐ 6
☐ 7

5. If I could live my life over, I would change almost nothing.

☐ 1

☐ 2

☐ 3

☐ 4

☐ 5

☐ 6

☐ 7

Flourishing Scale (FS)

Instructions:

Below are eight statements that you may agree or disagree with. Indicate your agreement with each item. (1 = Strongly Disagree, 7 = Strongly Agree)

1. I lead a purposeful and meaningful life.

☐ 1

☐ 2

☐ 3

☐ 4

☐ 5

☐ 6

☐ 7

2. My social relationships are supportive and rewarding.

☐ 1

☐ 2

☐ 3

☐ 4

☐ 5

☐ 6

☐ 7

3. I am engaged and interested in my daily activities.

☐ 1

☐ 2

☐ 3

☐ 4

☐ 5

☐ 6

☐ 7

4. I actively contribute to the happiness and well-being of others.

☐ 1

☐ 2

☐ 3

☐ 4

☐ 5

☐ 6

☐ 7

5. I am competent and capable in the activities that are important to me.

☐ 1
☐ 2
☐ 3
☐ 4
☐ 5
☐ 6
☐ 7

6. I am a good person and live a good life.

☐ 1
☐ 2
☐ 3
☐ 4
☐ 5
☐ 6
☐ 7

7. I am optimistic about my future.

☐ 1
☐ 2
☐ 3
☐ 4
☐ 5
☐ 6
☐ 7

8. People respect me.

☐ 1
☐ 2
☐ 3
☐ 4
☐ 5
☐ 6
☐ 7

Dog-Owner Relationship Scale (DORS-28)

Instructions:
Please indicate how true each statement is for you, or how frequently each behaviour occurs, using the response options provided beneath each item.

1. How often does your dog stop you doing things you want to?

☐ Never
☐ Almost never
☐ Occasionally
☐ More than occasionally
☐ Often

☐ Very often
☐ Extremely often

2. How often do you kiss your dog?

☐ Never
☐ Almost never
☐ Occasionally
☐ More than occasionally
☐ Often
☐ Very often
☐ Extremely often

3. My dog provides me with constant companionship.

☐ Strongly disagree
☐ Disagree
☐ Somewhat disagree
☐ Neither agree nor disagree
☐ Somewhat agree
☐ Agree
☐ Strongly agree

4. How often do you take your dog in the car, on your bike, or on public transport?

☐ Never
☐ Almost never
☐ Occasionally
☐ More than occasionally
☐ Often
☐ Very often
☐ Extremely often

5. It bothers me that my dog stops me doing things I enjoyed before I owned it.

☐ Strongly disagree
☐ Disagree
☐ Somewhat disagree
☐ Neither agree nor disagree
☐ Somewhat agree
☐ Agree
☐ Strongly agree

6. How often do you hug your dog?

☐ Never
☐ Almost never
☐ Occasionally
☐ More than occasionally
☐ Often
☐ Very often
☐ Extremely often

7. My dog is there whenever I need to be comforted.

☐ Strongly disagree
☐ Disagree
☐ Somewhat disagree
☐ Neither agree nor disagree
☐ Somewhat agree
☐ Agree
☐ Strongly agree

8. How often do you take your dog to visit people?

☐ Never
☐ Almost never
☐ Occasionally
☐ More than occasionally
☐ Often
☐ Very often
☐ Extremely often

9. It is annoying that sometimes I have to change my plans because of my dog.

☐ Strongly disagree
☐ Disagree
☐ Somewhat disagree
☐ Neither agree nor disagree
☐ Somewhat agree
☐ Agree
☐ Strongly agree

10. How often do you cuddle your dog?

☐ Never
☐ Almost never
☐ Occasionally
☐ More than occasionally
☐ Often
☐ Very often
☐ Extremely often

11. My dog is constantly attentive to me.

☐ Strongly disagree
☐ Disagree
☐ Somewhat disagree
☐ Neither agree nor disagree
☐ Somewhat agree
☐ Agree
☐ Strongly agree

12. How often do you groom your dog?

☐ Never
☐ Almost never
☐ Occasionally
☐ More than occasionally
☐ Often
☐ Very often
☐ Extremely often

13. My dog costs too much money.

☐ Strongly disagree
☐ Disagree
☐ Somewhat disagree
☐ Neither agree nor disagree
☐ Somewhat agree
☐ Agree
☐ Strongly agree

14. How often do you tell your dog things you do not tell anyone else?

☐ Never
☐ Almost never
☐ Occasionally
☐ More than occasionally
☐ Often
☐ Very often
☐ Extremely often

15. How often do you feel that looking after your dog is a chore?

☐ Never
☐ Almost never
☐ Occasionally
☐ More than occasionally
☐ Often
☐ Very often
☐ Extremely often

16. How traumatic do you think it will be for you when your dog dies?

☐ Not at all traumatic
☐ A little traumatic
☐ Somewhat traumatic
☐ Quite traumatic
☐ Very traumatic
☐ Extremely traumatic
☐ Unbearably traumatic

17. How often do you pet your dog?

☐ Never
☐ Almost never
☐ Occasionally
☐ More than occasionally
☐ Often
☐ Very often
☐ Extremely often

18. If everyone else left me, my dog would still be there for me.

☐ Strongly disagree
☐ Disagree
☐ Somewhat disagree
☐ Neither agree nor disagree
☐ Somewhat agree
☐ Agree
☐ Strongly agree

19. My dog makes too much mess.

☐ Strongly disagree
☐ Disagree
☐ Somewhat disagree
☐ Neither agree nor disagree
☐ Somewhat agree
☐ Agree
☐ Strongly agree

20. How often do you play games with your dog?

☐ Never
☐ Almost never
☐ Occasionally
☐ More than occasionally
☐ Often
☐ Very often
☐ Extremely often

21. How often do you feel that having your dog is more trouble than it is worth?

☐ Never
☐ Almost never
☐ Occasionally
☐ More than occasionally
☐ Often
☐ Very often
☐ Extremely often

22. How often do you talk to your dog?

☐ Never
☐ Almost never
☐ Occasionally
☐ More than occasionally
☐ Often
☐ Very often
☐ Extremely often

23. My dog helps me get through tough times.

☐ Strongly disagree
☐ Disagree
☐ Somewhat disagree
☐ Neither agree nor disagree
☐ Somewhat agree
☐ Agree
☐ Strongly agree

24. How often do you buy your dog gifts?

☐ Never
☐ Almost never
☐ Occasionally
☐ More than occasionally
☐ Often
☐ Very often
☐ Extremely often

25. How difficult is it to look after your dog?

☐ Very easy
☐ Easy
☐ Somewhat easy
☐ Neither difficult nor easy
☐ Somewhat difficult
☐ Difficult
☐ Very difficult

26. How often do you spend time enjoying watching your dog?

☐ Never
☐ Almost never
☐ Occasionally
☐ More than occasionally
☐ Often
☐ Very often
☐ Extremely often

27. There are major aspects of owning a dog I do not like.

☐ Strongly disagree
☐ Disagree
☐ Somewhat disagree
☐ Neither agree nor disagree
☐ Somewhat agree
☐ Agree
☐ Strongly agree

28. How often do you give your dog food treats?

☐ Never
☐ Almost never
☐ Occasionally
☐ More than occasionally
☐ Often
☐ Very often
☐ Extremely often

Supplementary Materials

S2: Simple Slopes for Secondary Moderation Models

Figure S1. *Voluntariness × Human–Dog Bond (DORS-28) Predicting Life Satisfaction (SWLS)*

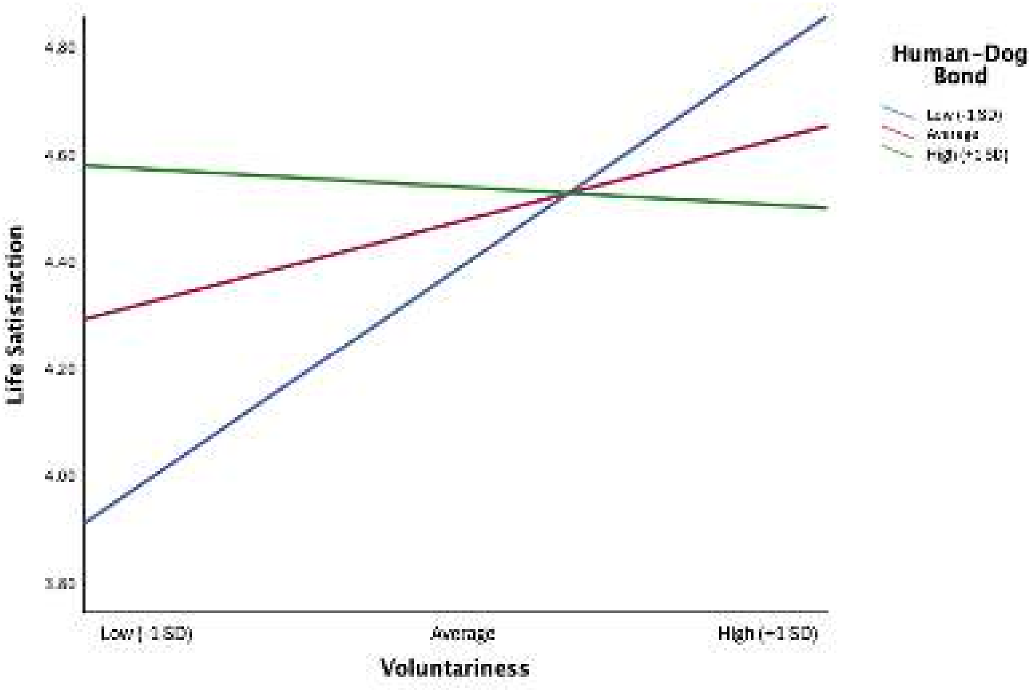

Note. Predicted values are plotted at low (–1 SD), mean, and high (+1 SD) levels of voluntariness and the human–dog bond (DORS-28 total), controlling for parental status (parent vs non-parent), age, education, income and relationship status, estimated from moderated regression (PROCESS) with mean-centred variables.

Figure S2. *Voluntariness × Human–Dog Bond (DORS-28) Predicting Flourishing (FS)*

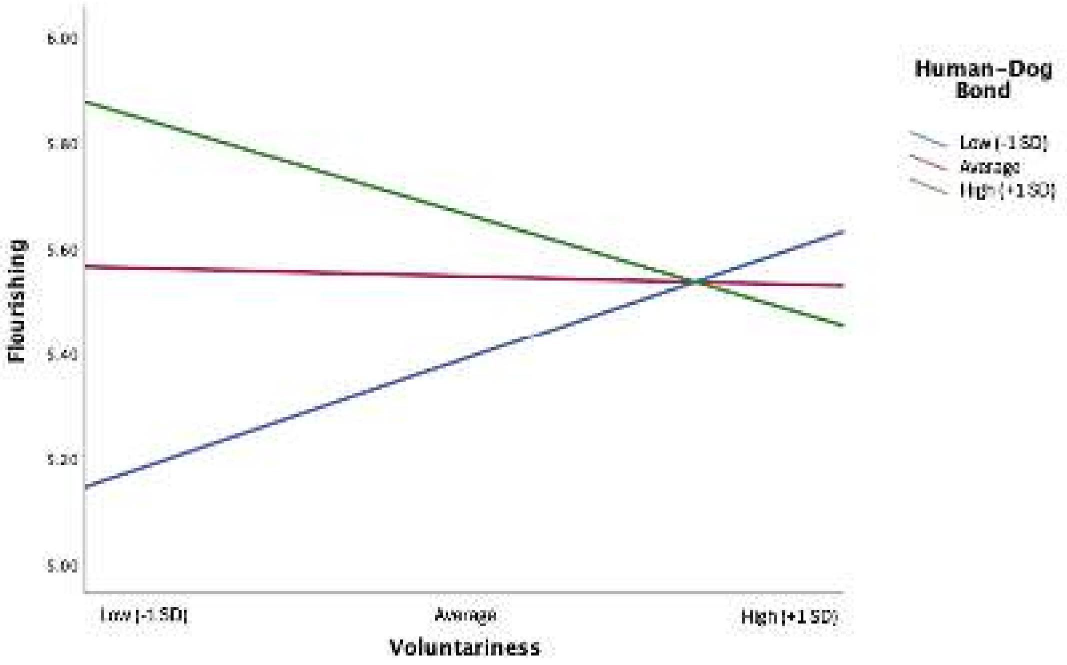

Note. Predicted values are plotted at low (–1 SD), mean, and high (+1 SD) levels of voluntariness and the human–dog bond (DORS-28 total), controlling for parental status (parent vs non-parent), age, education, income and relationship status, estimated from moderated regression (PROCESS) with mean-centred variables.
